# Longitudinal changes of blood β-synuclein in cognitively unimpaired, mild cognitive impairment and sporadic Alzheimer´s disease

**DOI:** 10.1186/s13195-026-01973-1

**Published:** 2026-02-11

**Authors:** Patrick Oeckl, Samir Abu-Rumeileh, Christopher M. Weise, Markus Otto, Alzheimer’s Disease Neuroimaging Initiative

**Affiliations:** 1 Department of Neurology, Ulm University Hospital, Helmholtzstr. 8/1, Ulm, 89081 Germany; 2 German Center for Neurodegenerative Diseases (DZNE) Ulm, Helmholtzstr. 8/1, Ulm, 89081 Germany; 3 Department of Neurology, Martin-Luther-University Halle-Wittenberg, Ernst-Grube-Str. 40, Halle (Saale), 06120 Germany

**Keywords:** β-synuclein, Blood biomarker, Sporadic Alzheimer´s disease, Mild cognitive impairment, Longitudinal observation, Synaptic degeneration

## Abstract

**Background:**

β-Synuclein is an emerging synaptic blood biomarker for Alzheimer´s disease (AD) and correlates with cognitive impairment, brain atrophy and amyloid/tau pathology. Longitudinal data from individual patients are missing so far but are important to evaluate how changes of β-synuclein might be used in early diagnosis, prediction, disease progression and treatment monitoring.

**Methods:**

In this observational study, we investigated serum β-synuclein by immunoprecipitation-mass spectrometry (IP-MS) in 463 participants from the Alzheimer’s Disease Neuroimaging Initiative (ADNI) including clinically diagnosed cognitively unimpaired, mild cognitive impairment (MCI) and AD dementia subjects with ≥ 1 follow-up samples for 235 individuals and clinical follow-up for up to 19 years. CSF AD biomarker levels were available for 194 participants.

**Results:**

Participants (40.0% female, *n* = 185) had a mean (± SD) age of 76.2 ± 6.7 years. The cross-sectional group comparison yielded higher β-synuclein levels in MCI and AD dementia compared with CU and in AD dementia vs MCI patients. Mean follow-up time of longitudinal serum samples was 2.3 ± 1.2 years. The longitudinal data indicate that β-synuclein levels are dynamic during all stages of the AD continuum (CU, MCI, dementia) with substantial inter-individual variation. β-Synuclein predicted MCI-to-dementia conversion and future cognitive decline and it performed better in discrimination of AD dementia patients than CSF neurogranin.

**Conclusions:**

Our longitudinal data support the use of serum β-synuclein levels for prediction of future cognitive decline and MCI-to-dementia conversion but needing confirmation. Further studies with biologically and clinically defined participants must verify the trajectories of β-synuclein during the AD continuum.

**Supplementary Information:**

The online version contains supplementary material available at 10.1186/s13195-026-01973-1.

## Background

β-Synuclein is a presynaptic protein and has emerged as a promising blood biomarker candidate to track synaptic changes in Alzheimer´s disease (AD). Cross-sectional studies showed higher levels of β-synuclein in sporadic AD patients and correlation with cognitive impairment, temporal brain atrophy and amyloid and tau-pathology [1–4]. Elevated levels were even observed in early AD cases with mild cognitive impairment (MCI) and preclinical AD and indicated that synaptic changes start after amyloid deposition but before tau accumulation [3, 4]. In individuals with Down Syndrome, a genetic form of AD, blood β-synuclein levels are higher in subjects without clinical signs of AD, consistent with the preclinical increase in sporadic AD [5].

We recently provided first temporal information about the timepoint when β-synuclein levels start to rise in the blood by investigating asymptomatic autosomal dominant AD (ADAD) mutation carriers from the Dominantly Inherited Alzheimer Network (DIAN) [6]. The data indicated that β-synuclein starts to increase approximately 11 years before symptom onset thereby preceeding axonal neurodegeneration, cognitive symptoms, brain atrophy and hypometabolism. So there is strong evidence from these cross-sectional data that β-synuclein in blood might be suitable to detect synaptic degeneration during the diagnostic work-up and monitoring of preclinical AD. In the context of the recent success with anti-amyloid drugs and the possibility to slow down disease progression, the strategy for presymptomatic diagnosis and follow-up is one of the most important questions in the AD field. In addition, trials with different anti-amyloid antibodies indicated that synaptic markers could be promising outcome measures to monitor positive treatment effects [7–9]. In fact, treatment led to a lowering of CSF levels of the synaptic marker neurogranin (NRGN), a marker that shows strong correlation with CSF β-synuclein [10]. Measurement of β-synuclein in blood would have many advantages regarding accessibility, costs and repeated sampling.

To evaluate the potential use of β-synuclein as a marker in early diagnosis, prediction, disease progression and treatment monitoring, it is important to define the longitudinal changes in individual subjects and during the AD continuum. No such data are available today. The intra-individual dynamics over time of β-synuclein levels will be an important indicator for its applicability to monitor changes preclinically and during treatment. It will be a basis for power calculations and sample size estimation when used as an outcome measure in clinical trials. Longitudinal data are also required to validate the temporal assumptions from cross-sectional studies and to translate findings from ADAD mutation carriers to sporadic AD.

The aim of the present study was to characterize serum β-synuclein levels in longitudinal samples from participants of the Alzheimer´s Disease Neuroimaging Initiative (ADNI) at different stages of AD and with clinical follow-up data for up to 19 years enabling an exact temporal placement in the AD continuum and evaluating the predictive value of β-synuclein measurements. In addition, we compared serum β-synuclein with CSF NRGN.

## Methods

Data used in the preparation of this article were obtained from the ADNI database (adni.loni.usc.edu). The ADNI was launched in 2003 as a public–private partnership, led by Principal Investigator Michael W. Weiner, MD. The primary goal of ADNI has been to test whether serial magnetic resonance imaging (MRI), positron emission tomography (PET), other biological markers, and clinical and neuropsychological assessment can be combined to measure the progression of MCI and early AD.

### Participants

In our study, we included serum samples from 463 ADNI participants that were recruited between 2005–2011 and for 235 individuals, at least one follow-up serum sample was available (see Fig. S1). The participants had the following clinical diagnosis at the baseline visit (i.e. time of sample collection of the first visit available in our study): 135 cognitively unimpaired (CU) subjects, 166 MCI and 162 AD dementia patients. Aβ-positivity (Aβ+) was defined using CSF Aβ42 concentration and a cut-off of 980 pg/ml based on the related method description in the ADNI database. We used the Mini-Mental State Examination (MMSE) and clinical dementia rating sum-of-boxes (CDR-SB) as measures for the cognitive status. Characteristic of patients are listed in Table 1.

**Table 1:** Demographic and clinical characteristics of participants

	**CU** **(*n* = 135)**		**MCI** **(*n* = 166)**	**AD** **(*n* = 162)**	***p*-value**
No. of females	65 (48.1%)		55 (33.1%)	65 (40.1%)	*p* = 0.03
Age (years)^a^	75.6 (72.5–78.7)		77.1 (72.3–82.0)	76.8 (71.4–81.8)	*p* = 0.38
Education (years)^a^	16 (14–18)		16 (13–18)	16 (13–18)^b^	*p* < 0.05
No. of ApoE4 positives^a^	38 (28.1%)		84 (50.6%)	104 (64.2%)	*p* < 0.0001
Dementia severity				Mild (*n* = 145)	
				Mod (*n* = 16)	
				Sev (*n* = 1)	
MMSE^a^	29 (29–30)		28 (26–29)^c^	23 (21–25)^c,d^	*p* < 0.0001
CDR-SB^a^	0 (0–0)		1.5 (1.0–2.5)^c^	4.5 (3.5–5.5)^c,d^	*p* < 0.0001
Serum β-synuclein (pg/mL)^a^	8.32 (6.97–10.6)		10.7 (8.28–13.7)^c^	13.3 (10.7–16.5)^c,d^	*p* < 0.0001
	**CU (Aβ-)**	**CU (Aβ+)**	**MCI (Aβ+)**	**AD (Aβ+)**	
	**(*n* = 40)**	**(*n* = 28)**	**(*n* = 39)**	**(*n* = 64)**	
No. of females	17 (42.5%)	14 (50.0%)	17 (43.6%)	25 (39.1%)	*p* = 0.81
Age (years)^a^	73.6 (71.2–77.9)	76.7 (73.3–78.2)	75.1 (71.6–79.2)	75.0 (70.0–80.0)	*p* = 0.64
Education (years)^a^	17 (15–18)	16 (16–18)	17 (14–18)	16 (13–18)	*p* = 0.11
No. of ApoE4 positives^a^	6 (15.0%)	13 (46.4%)	24 (61.5%)	48 (75.0%)	*p* < 0.0001
MMSE^a^	29 (29–30)^e,f^	30 (29–30)^e,f^	27 (26–29)^f^	24 (22–25)	*p* < 0.0001
CDR-SB^a^	0 (0–0)^f,g^	0 (0–0)^f,g^	1.5 (1.0–2.0)^f^	4.0 (3.5–5)	*p* < 0.0001
Serum β-synuclein (pg/mL)^a^	7.58^f,h^ (6.52–9.79)	9.32^f,i^ (7.13–11.3)	11.8 (9.42–14.6)	13.3 (10.5–16.3)	*p* < 0.0001

*AD* Alzheimer´s disease, *CDR-SB* Clinical dementia rating sum-of-boxes, *CU* Cognitively unimpaired, *MCI* Mild cognitive impairment, *MMSE* Mini-Mental State Examination, *Mod* Moderate, *Sev* Severe

^a^median and interquartile range

^b^*p* < 0.05 vs CU

^c^*p* < 0.0001 vs CU

^d^*p* < 0.0001 vs MCI

^e^*p* < 0.01 vs MCI (Aβ+)

^f^*p* < 0.0001 vs AD (Aβ+)

^g^*p* < 0.0001 vs MCI (Aβ+)

^h^*p* < 0.001 vs MCI (Aβ+)

^i^*p* < 0.05 vs MCI (Aβ+)

The longitudinal follow-up procedure in ADNI 1 and ADNI GO comprised regular visits every 6 months including clinical tests and blood collection. For our study, we received serum samples from yearly visits. For more details, we refer to the ADNI database (adni.loni.usc.edu).

### Determination of serum β-synuclein by IP-MS

Serum β-synuclein was measured in our lab by immunoprecipitation mass spectrometry (IP-MS) as previously described [2]. In brief, 490µL aliquots of serum were mixed with an internal standard solution containing recombinant ^15^N-β-synuclein (rPeptide, Watkinsville, GA, USA) and immunoprecipitated using magnetic beads coupled with an anti-β-synuclein antibody (EP1537Y from Abcam, Cambridge, UK). Beads were washed with 50 mM triethylammonium bicarbonate/0.1% n-Dodecyl-β-D-maltoside using a KingFisher Apex instrument and eluted. β-Synuclein was digested by trypsin/LysC (Promega, Walldorf, Germany) and two proteotypic peptides were quantified by LC-MRM (aa46-58 and aa61-85) using an Eksigent MicroLC200, Agilent 1260 pump and Sciex QTRAP6500 mass spectrometer in multiple reaction monitoring (MRM) mode. Calibrators were prepared using recombinant human fulllength β-synuclein (without tags) from rPeptide and the exact concentration of the β-synuclein stock solution was quantified by amino acid analysis (Alphalyse A/S, Odense, Denmark). Calibration range was 2 to 30 pg/mL. Samples were analyzed in a total of 14 runs and serum quality control (QC) samples (low, medium, high) were included in all runs. The deviation of the QC samples from the reference value (i.e. the mean over all measurements) was monitored and must be ≤ 15% (deviation of individual QC values up to 20% were accepted). Intraassay CVs were in the range of 0.5–11.5% and interassay CV 8.8–11.1%. Samples were randomly assigned to the different runs and the analysts were blinded to the patient data.

### Determination of CSF biomarkers

Data for additional CSF and plasma biomarkers were extracted from the ADNI database. CSF AD core biomarkers Aβ42, tau protein phosphorylated at S181 (pTau181) and total Tau (tTau) were measured by the Roche Elecsys immunoassays at UPenn. CSF NRGN was measured at UGot by a homemade MSD assay. CSF (ELISA) and plasma (Simoa) neurofilament light chain (NfL) were also measued at UGot. More details on the measurement procedures are available in the ADNI database (https://adni.loni.usc.edu).

### Statistics

We used SPSS 29.0.1.0 and GraphPad Prism 8.3.0 for the statistical analysis. Normal distribution of variables in the diagnostic groups was tested by the Shapiro Wilk test. Categorical variables were compared by the Chi-square test. Non-parametric tests were used for group comparisons of non-normally distributed variables such as age, years of education, MMSE, CDR-SB and β-synuclein rate-of-change (RoC). Serum β-synuclein levels were log10 tranformed to receive a normal distribution and groups were compared by univariate general linear regression including age, sex, ApoE4-positivity and education as covariates. The individual annual β-synuclein RoC was calculated using linear regression (slope) across all available time points (minimum of one follow-up sample, mean follow-up time was 2.3 ± 1.2yrs and maximum 4.5yrs). Annual RoC for MMSE and CDR-SB was calculated likewise. Normalized RoC (%RoC) is the RoC value relative to the first visit. We used linear mixed-effects models to investigate the interaction of time with other variables. Correlation analyses were performed using the Spearman rank correlation coefficient (r_s_). Receiver operating characteristic (ROC) curve analysis including the area under the curve (AUC) was used to assess the discriminatory performance of biomarkers. A p-value < 0.05 was regarded significant.

## Results

### Participants

Our study included 463 participants (40.0% female, *n* = 185) from the ADNI cohort (ADNI 1 and ADNI GO) with a mean (± SD) age of 76.2 ± 6.7 years. Characteristics of participants are given in Table 1. Age was not different between diagnostic groups (*p* = 0.38, Table 1) but there was a significant difference of sex distribution (*p* = 0.03) and ApoE4 positivity (*p* < 0.0001). AD dementia cases had less years of education compared with CU (*p* < 0.05) and cognitive scores were increasingly abnormal in MCI and AD dementia (*p* < 0.0001, Table 1).

We observed a moderate correlation of serum β-synuclein with age in CU (r_s_ = 0.29, *p* < 0.001) but not in MCI (r_s_ = 0.03, *p* = 0.67), AD (r_s_ = −0.09, *p* = 0.23) and the whole cohort (r_s_ = 0.05, *p* = 0.25). Serum β-synuclein levels were slightly higher in female participants in the CU group (median 8.49 IQR 7.57–10.7 vs. 7.73 IQR 6.17–10.3 in male) but this was not significant after adjusting for age, MMSE, CDR-SB, education and ApoE4-positivity (*p* = 0.17). ApoE4-positivity did not have an effect on serum β-synuclein levels in CU (*p* = 0.10) after adjusting for age, sex, MMSE, CDR-SB and education.

### Cross-sectional group comparisons of serum β-synuclein levels

**Fig. 1:**
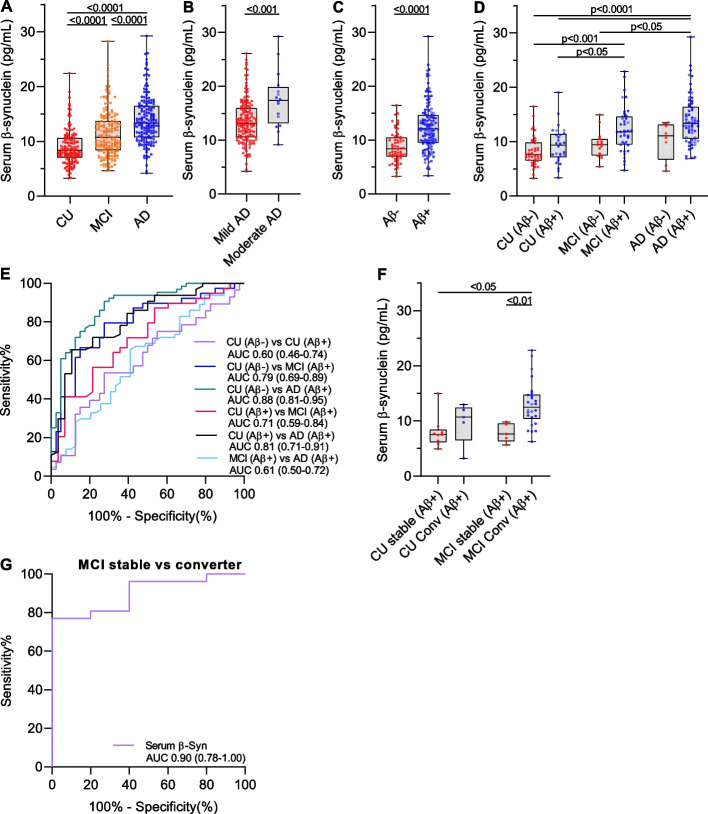
Group comparisons of serum β-synuclein levels and discriminative performance. Serum β-synuclein levels were compared between (**A**) cognitively unimpaired (CU, *n* = 135), mild cognitive impairment (MCI, *n* = 166) and Alzheimer´s disease dementia (AD, *n* = 162) participants, (**B**) patients with mild (*n* = 145) and moderate AD (*n* = 16), (**C**) Aβ+ (*n* = 131) and Aβ- (*n* = 63) subjects and (**D**) in the diagnostic groups stratified according to Aβ status. **E** Receiver operating characteristic (ROC) curves for the discrimination of CU (Aβ- and Aβ+), MCI (Aβ+) and AD dementia (Aβ+) participants by serum β-synuclein levels. **F** CU (Aβ+) participants and patients with MCI (Aβ+) with clinical follow-up data were stratified into stable CU (> 5yrs, *n* = 9) and MCI (> 5yrs, *n* = 5) and converters from CU-to-MCI/dementia (< 5yrs, *n* = 5) and MCI-to-dementia (< 5yrs, *n* = 26). **G** ROC curve for the discrimination of stable MCI (Aβ+) and MCI (Aβ+) converters by serum β-synuclein levels. In box plots, the dots are individual values, boxes are median and interquartile range, whiskers are min and max. Groups were compared using log10 tranformed β-synuclein values and general linear regression including age, sex, education and ApoE4 status as covariates. In ROC curves, the area under the curve (AUC) is given and the 95% confidence interval is indicated in brackets

We observed significantly higher serum β-synuclein levels in participants with MCI (median 10.7 pg/mL, IQR 8.28–13.7, *p* < 0.0001) and AD dementia (13.3 pg/mL, IQR 10.7–16.5, *p* < 0.0001) compared with CU (8.32 pg/mL, IQR 6.97–10.6) and in AD dementia compared with MCI (*p* < 0.0001, Fig. 1A). Staging of AD dementia into mild and moderate revealed higher β-synuclein levels in moderate (17.4 pg/mL, IQR 13.1–19.9, *n* = 16) compared with mild AD dementia (13.1 pg/mL, IQR 10.5–16.0, *n* = 145, *p* < 0.001, Fig. 1B).

CSF Aβ42 levels were available for 194 participants (68 CU, 54 MCI, 72 AD dementia) and we stratified them into Aβ+ (*n* = 131) and Aβ- (*n* = 63, threshold 980 pg/mL). Serum β-synuclein levels were higher in Aβ+ (12.0 pg/mL, IQR 9.42–14.6) than Aβ- individuals (8.35 pg/mL, IQR 6.87–10.5, *p* < 0.0001, Fig. 1C). Stratification of the diagnostic groups according to Aβ-positivity revealed significantly higher serum β-synuclein levels only in Aβ-positive MCI and AD dementia compared with CU (Fig. 1D) but not Aβ- subjects indicating strong association of elevated β-synuclein levels with amyloid-related pathology.

Serum β-synuclein discriminated CU (Aβ-)/CU (Aβ+) from AD dementia (Aβ+) with an AUC of 0.88/0.81 and a sensitivity and specificity of 90.6%/65.6% and 72.5%/89.3% at a threshold of 8.97/12.1 pg/mL (Fig. 1E). The AUCs for discrimination of CU (Aβ-)/CU (Aβ+) vs MCI (Aβ+) and MCI (Aβ+) vs AD dementia (Aβ+) were 0.79/0.71 and 0.61 (Fig. 1E).

### Predictive value of serum β-synuclein levels

Follow-up data (≥ 6 months) for the clinical diagnosis of CU and MCI participants was available for 282 participants (126 CU, 156 MCI, see Fig. S1) with a mean follow-up time of 6.1 ± 4.4yrs (CU 7.6 ± 5.1yrs, MCI 4.8 ± 3.3yrs) and a maximum of 18.8yrs. 14 CU participants converted to the MCI or dementia stage within 5 years whereas 66 CU were stable for at least 5 years. The remaining 46 CU were stable during the follow-up but follow-up time was less than 5 years. 71 MCI participants converted to dementia within 5 years whereas 35 MCI were stable for at least 5 years (Fig. S1). The remaining MCI patients were stable during the follow-up but with a follow-up time < 5 years (*n* = 44) or were diagnosed as CU during follow-up (*n* = 6).

Considering only those participants with available Aβ-status and being Aβ+, 5 CU (Aβ+) converted to the MCI or dementia stage within 5 years whereas 9 CU (Aβ+) were stable for at least 5 years. In the MCI (Aβ+) group, 26 converted to the dementia stage within 5 years whereas 5 where stable. Serum β-synuclein levels were higher in MCI (Aβ+) converters (12.6 pg/mL, IQR 10.4–14.8) compared with stable MCI (Aβ+) (7.67 pg/mL, IQR 6.37–9.65, *p* < 0.01) and stable CU (Aβ+) (7.57 pg/mL, IQR 6.19–8.52, *p* < 0.05) but not different between the other groups (Fig. 1F). β-Synuclein discriminated MCI (Aβ+) converters from stable MCI (Aβ+) with an AUC of 0.90 (95% CI 0.78–1.00, Fig. 1G) and a sensitivity and specificity of 76.9% and 100% (threshold 10.3 pg/mL).

We evaluated the predicitve value of baseline serum β-synuclein levels on future cognitive decline by investigating 409 participants with at least one follow for MMSE and CDR-SB (maximum 5 years). Serum β-synuclein significantly correlated with the MMSE and CDR-SB at baseline and predicted cognitive status at follow-up visits at 1–5 years (MMSE: r_s_ = −0.393 to −0.502, CDR-SB: r_s_ = 0.422 to 0.490, *p* < 0.0001, Table 2). Notably, β-synuclein predicted the annual RoC of MMSE and CDR-SB (r_s_ = −0.352 and 0.41, *p* < 0.0001). When stratified according to diagnostic groups, β-synuclein showed highest correlations with MMSE and CDR-SB in MCI patients and especially predicted them at years 4 (r_s_ = −0.582 and 0.406) and 5 (r_s_ = −0.431 and 0.470) compared with baseline (r_s_ = −0.316 and 0.195) and also with the RoC of both scores. No significant correlations were observed in CU and AD dementia participants (Table 2).

**Table 2:** Association of baseline serum β-synuclein levels with future cognitive decline

	MMSE			CDR-SB		
	r_s_-value	*p*-value	N	r_s_-value	*p*-value	N
**All**						
BL	**−0.450**	**9.09E-22**	409	**0.468**	**1.27E-23**	409
Year 1	**−0.472**	**1.11E-22**	383	**0.486**	**4.95E-24**	382
Year 2	**−0.517**	**1.48E-21**	295	**0.490**	**1.01E-20**	320
Year 3	**−0.393**	**8.29E-09**	200	**0.443**	**1.19E-11**	213
Year 4	**−0.502**	**8.66E-10**	132	**0.422**	**3.66E-08**	157
Year 5	**−0.447**	**1.38E-07**	127	**0.443**	**4.31E-08**	140
RoC	**−0.352**	**2.28E-13**	409	**0.410**	**5.37E-18**	409
**AD**						
BL	−0.074	4.08E-01	127	0.056	5.30E-01	127
Year 1	−0.165	7.11E-02	120	0.135	1.42E-01	120
Year 2	−0.114	2.86E-01	90	0.123	2.32E-01	96
Year 3	**0.522**	**4.79E-02**	15	−0.185	4.23E-01	21
Year 4	−0.422	2.98E-01	8	−0.046	8.97E-01	11
Year 5	0.400	7.50E-01	4	−0.030	1.00E + 00	6
RoC	−0.147	9.91E-02	127	**0.208**	**1.88E-02**	127
**MCI**						
BL	**−0.316**	**5.76E-05**	156	**0.195**	**1.48E-02**	156
Year 1	**−0.330**	**5.82E-05**	143	**0.256**	**2.15E-03**	142
Year 2	**−0.485**	**2.02E-07**	103	**0.292**	**1.56E-03**	115
Year 3	**−0.391**	**2.01E-04**	86	**0.415**	**2.84E-05**	95
Year 4	**−0.582**	**1.35E-06**	59	**0.406**	**2.30E-04**	78
Year 5	**−0.431**	**3.02E-04**	66	**0.470**	**5.15E-05**	68
RoC	**−0.360**	**3.84E-06**	156	**0.295**	**1.88E-04**	156
**CU**						
BL	0.031	7.30E-01	126	0.101	2.61E-01	126
Year 1	−0.102	2.67E-01	120	0.093	3.13E-01	120
Year 2	−0.138	1.66E-01	102	0.037	7.04E-01	109
Year 3	−0.028	7.82E-01	99	0.048	6.39E-01	97
Year 4	−0.120	3.42E-01	65	−0.048	6.96E-01	68
Year 5	−0.222	9.73E-02	57	0.136	2.77E-01	66
RoC	−0.094	2.93E-01	126	0.070	4.37E-01	126

Correlation analyses were performed using Spearmans rank correlation coefficient (r_s_). Significant correlations are indicated in bold

*AD* Alzheimer´s disease, *BL* Baseline visit, *CDR-SB* Clinical dementia rating sum-of-boxes, *CU* Cognitively unimpaired, *MCI* Mild cognitive impairment, *MMSE* Mini-Mental State Examination, *RoC* Rate-of-change

### Longitudinal changes of serum β-synuclein

**Fig. 2:**
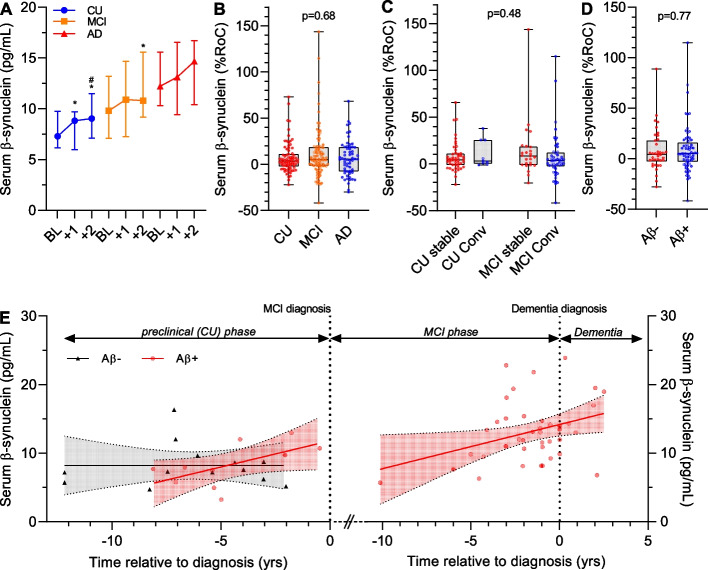
Longitudinal changes of serum β-synuclein. **A** β-synuclein levels at first visit (BL), year 1 (+ 1) and year 2 (+ 2) from cognitively unimpaired (CU, *n* = 20), mild cognitive impairment (MCI, *n* = 29) and Alzheimer´s disease dementia (AD, *n* = 20) participants with two annual follow-up samples. Data show the median and interquartile range and were compared using general linear regression for repeated measures including age, sex, education and ApoE4 status as covariates. *p < 0.05 to BL, ^#^*p* < 0.05 to (+ 1). **B**-**D** Serum β-synuclein rate-of-change (%RoC) in (**B**) CU (*n* = 78), MCI (*n* = 96) and AD dementia (*n* = 61) participants, (**C**) in stable CU (> 5yrs, *n* = 48) and MCI (> 5yrs, *n* = 25) and converters from CU-to-MCI/dementia (< 5yrs, *n* = 9) and MCI-to-dementia (< 5yrs, *n* = 49) and (**D**) Aβ- (*n* = 38) and Aβ+ (*n* = 68) participants. Dots are individual values, boxes are median and interquartile range, whiskers are min and max. Groups were compared by the Mann Whitney test or Kruskal Wallis test followed by Dunn´s posthoc test. No significant differences were observed. **E** Serum β-synuclein levels in CU (Aβ-) (*n* = 13), CU (Aβ+) (*n* = 10), MCI (Aβ+) (*n* = 29) and AD dementia (Aβ+) (*n* = 11) participants relative to the time of MCI and dementia diagnosis. Dots are individual values. Solid lines are simple linear regression with the 95% confidence interval. Dotted lines indicate the time point of diagnosis defined as point zero

Annual follow-up samples were available for 235 participants (78 CU, 96 MCI, 61 AD dementia, see Fig. S1) with a mean follow-up time of 2.3 ± 1.2yrs (CU 3.2 ± 1.1yrs, MCI 2.1 ± 1.2yrs, AD dementia 1.6 ± 0.7yrs) and a maximum of 4.5yrs. To compare longitudinal changes between groups, we analyzed participants with at least two consecutive annual follow-up samples (20 CU, 29 MCI, 20 AD dementia). Serum β-synuclein levels increased in CU and MCI participants during follow-up whereas there was no significant change in the AD dementia group (Fig. 2A). We used the %RoC to compare longitudinal changes of serum β-Synuclein in the diagnostic groups. There was no significant difference of the %RoC between groups (*p* = 0.68, Fig. 2B), although the median %RoC was slightly higher in MCI (5.35%, IQR −1.58–18.6%) and AD dementia (5.60%, IQR −7.50–18.7%) compared with CU (3.60%, IQR −2.20–11.2%) but with high variation. The observation with absolute annual RoC values (Δpg/mL/year) was similar (CU: 0.29 pg/mL/year, IQR −0.20–0.79; MCI: 0.59 pg/mL/year, IQR −0.16–1.83; AD dementia: 0.72 pg/mL/year, IQR −1.14–1.94, *p* = 0.33). We also observed neither a significant difference of the %RoC between stable CU (4.40%, IQR −1.18–10.8%) and CU-to-MCI converter (3.10%, IQR 0.05–25.5%) nor between stable MCI (8.30%, IQR −1.20–18.4%) and MCI-to-dementia converter (3.50%, IQR −2.65–12.0%, *p* = 0.48, Fig. 2C). The β-synuclein %RoC was also not different between Aβ+ (5.00%, IQR −2.98–16.2%) and Aβ- (4.45%, IQR −2.53–18.3%, *p* = 0.77, Fig. 2D) participants. In addition to the %RoC, we also used linear mixed-effects models to estimate temporal differences of β-synuclein levels between groups. Similar to the %RoC analyses, there were no significant effects of time between groups (*p* = 0.47), stable CU/MCI and converters (*p* = 0.85) and between Aβ+ and Aβ- participants (*p* = 0.67).

For 174 participants, we had information about the time point of the first diagnosis of their current or the next AD disease stage (i.e. MCI or dementia). Figure 2E shows serum β-synuclein levels in a temporal relation to MCI and dementia diagnosis in CU (Aβ- and Aβ+), MCI (Aβ+) and AD dementia (Aβ+). β-Synuclein levels were most dynamic in the phase 3–5 years before dementia diagnosis.

### Comparison and correlation of serum β-synuclein with other fluid biomarkers

We compared β-synuclein levels with other fluid (CSF/plasma) biomarkers in a subset of patients including CSF Aβ42, pTau181, tTau, NRGN and plasma/CSF NfL. CSF NRGN, another synaptic fluid biomarker, significantly correlated with serum β-synuclein (r_s_ = 0.398, *p* < 0.0001) but showed stronger correlation with CSF tTau (r_s_ = 0.849) and pTau181 (r_s_ = 0.820) than serum β-synuclein (r_s_ = 0.422 and 0.453) whereas β-synuclein correlated stronger with CSF Aβ42 (r_s_ = −0.454 vs. −0.103) and cognitive impairment (MMSE: r_s_ = −0.467 vs. −0213, CDR-SB: r_s_ = 0.486 vs 0.345, Table 3). Serum β-synuclein showed also a moderate correlation with plasma NfL (r_s_ = 0.493), whereas with CSF NfL, the correlation was low (r_s_ = 0.202, Table 3).

**Table 3:** Correlation of serum β-synuclein with other fluid biomarkers

	Serum β-Syn	MMSE	CDR-SB	CSF Aβ42	CSF tTau	CSF pTau181	Plasma NfL	CSF NfL	CSF NRGN
Serum β-Syn		−0.467****	0.486****	−0.454****	0.422****	0.453****	0.493****	0.202*	0.398****
MMSE	*n* = 463		−0.783****	0.428****	−0.248***	−0.273***	−0.424****	−0.338****	−0.213**
CDR-SB	*n* = 461	*n* = 461		−0.493****	0.406****	0.422****	0.448****	0.427****	0.345****
CSF Aβ42	*n* = 194	*n* = 194	*n* = 194		−0.144*	−0.237***	−0.23**	−0.153	−0.103
CSF tTau	*n* = 194	*n* = 194	*n* = 194	*n* = 194		0.981****	0.167*	0.368****	0.849****
CSF pTau181	*n* = 194	*n* = 194	*n* = 194	*n* = 194	*n* = 194		0.169*	0.339****	0.82****
Plasma NfL	*n* = 248	*n* = 248	*n* = 247	*n* = 156	*n* = 156	*n* = 156		0.563****	0.108
CSF NfL	*n* = 155	*n* = 155	*n* = 155	*n* = 152	*n* = 152	*n* = 152	*n* = 154		0.167*
CSF NRGN	*n* = 148	*n* = 148	*n* = 148	*n* = 145	*n* = 145	*n* = 145	*n* = 147	*n* = 148	

Correlation analyses were performed using Spearmans rank correlation coefficient. *p < 0.05, **p < 0.01, ***p < 0.001, ****p < 0.0001

*CDR-SB* Clinical dementia rating sum-of-boxes, *MMSE* Mini-Mental State Examination, *pTau181*, tau protein phosphorylated at S181, *tTau* total tau protein, *NfL* Neurofilament light chain, *NRGN* Neurogranin

**Fig. 3:**
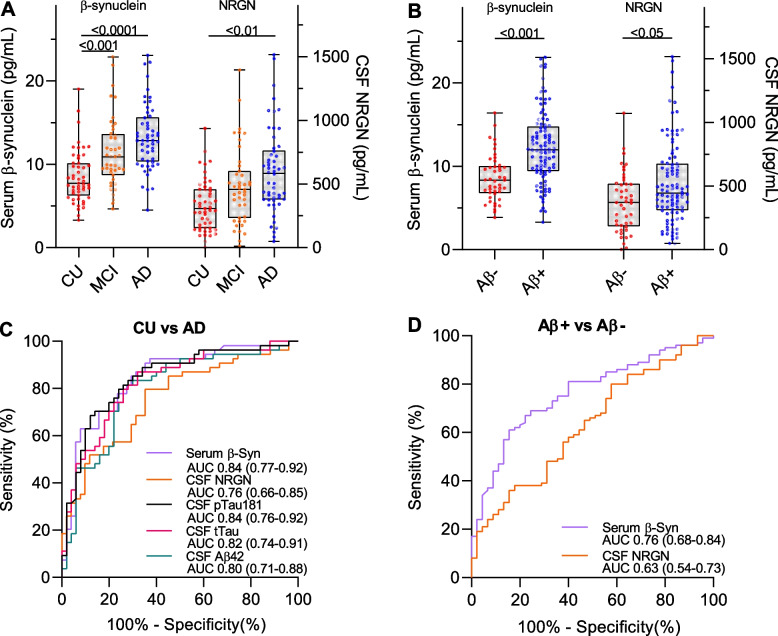
Comparison of serum β-synuclein with CSF neurogranin (NRGN). **A** Serum β-synuclein (β-Syn) and CSF NRGN levels in cognitively unimpaired (CU, *n* = 51), mild cognitive impairment (MCI, *n* = 43) and Alzheimer´s disease dementia (AD, *n* = 54) participants and (**B**) in Aβ+ (*n* = 100) and Aβ- (*n* = 45) subjects. Dots are individual values, boxes are median and interquartile range, whiskers are min and max. Groups were compared using log10 tranformed β-synuclein and NRGN values and general linear regression including age, sex, education and ApoE4 status as covariates. **C**, **D** Receiver operating characteristic (ROC) curves for the discrimination of (**C**) CU and AD dementia participants and (**D**) Aβ+ and Aβ- subjects. The area under the curve (AUC) is given for serum β-synuclein, CSF NRGN, CSF Aβ42, CSF tTau and CSF pTau181 and the 95% confidence interval is indicated in brackets

In the subgroup of participants with values for both β-synuclein and NRGN, both markers showed significantly higher levels in AD dementia and Aβ+ subjects and β-synuclein also in MCI (Fig. 3A, B). However, ROC curve analysis showed that β-synuclein better discriminated CU from AD dementia (AUC 0.84 vs 0.76) and Aβ+ from Aβ- participants (AUC 0.76 vs 0.63) than NRGN (Fig. 3C, D) with comparable discriminatory performance to the CSF AD core biomarkers Aβ42, tTau and pTau181.

## Discussion

In our study with 463 participants from ADNI, we provided for the first time longitudinal data on serum β-synuclein levels in CU and different disease stages of sporadic AD. The data indicate that β-synuclein levels are dynamic during all stages of the AD continuum with substantial inter-individual variation. β-Synuclein predicted MCI-to-dementia conversion and future cognitive decline and it performed better in discrimination of AD dementia patients than the synaptic CSF marker NRGN.

The definition of the longitudinal trajectories of blood β-synuclein levels in the AD continuum is of great importance to elucidate its value and potential applications in clinical routine or drug development. β-synuclein adds information about synaptic degeneration to the existing panel of established blood biomarkers such as Aβ42/40 (amyloid pathology), pTau variants (amyloid/tau pathology), NfL (axonal degeneration) or GFAP (neuroinflammation). β-synuclein shows strong correlation with pTau181 and GFAP but only moderate association with NfL, maybe due to the different mechanisms reflected [2, 11]. Markers such as β-synuclein and NfL are important to monitor the degeneration process in the brain which is required to evaluate treatment responses and detect the beginning of neurodegeneration in asymptomatic individuals.

We here provide data from 411 participants of the ADNI network with a clinical follow-up of up to 19 years and annual blood samples for up to 4.5 years enabling not only an exact temporal staging of the participants within the AD continuum but also the study of short-to-medium-term changes of β-synuclein in individual subjects. β-Synuclein levels were higher in follow-up samples of CU and MCI participants even after age-adjustment but not in AD dementia patients supporting an increase of β-synuclein over time flattening in the dementia stage. In agreement with this, temporal staging of participants indicated that β-synuclein levels seem to be most dynamic in the range of 3–5 years before dementia diagnosis. This is further supported by our observation that β-synuclein levels are elevated in MCI patients converting to the dementia stage within 5 years. On the other hand, we did not find a difference of the β-synuclein RoC in annual follow-up samples of CU, MCI and AD dementia participants that would explain elevated β-synuclein levels in MCI and AD dementia, although variation of RoC values was high. A similar RoC was also observed for stable MCI and MCI converters, although both groups significantly differed in the β-synuclein levels. An explanation could be that a quite sudden increase of β-synuclein in the medium-to-late MCI phase (3–5 yrs to dementia diagnosis) is responsible for most of the elevated β-synuclein levels in MCI and AD dementia which we could not detect because the individual follow-up time coverred by the blood samples in our study (mean 2.3yrs) was too short and temporally to late to capture enough participants at this time point to temporally resolve it. However, β-synuclein RoC varied strongly between participants even in stable CU which could also indicate that there are other confounding factors affecting serum β-synuclein levels. Further studies with follow-up samples over a longer timeframe and starting > 5 years before dementia diagnosis are needed to confirm this hypothesis and define the exact time point of rise. In addition, more studies on potential confounders for blood β-synuclein are required to reduce inter-individual variation of values.

Our data indicate that serum β-synuclein levels might predict future cognitive decline especially in MCI patients. In addition, β-synuclein predicted MCI-to-dementia conversion within 5 years. Thus, our longitudinal data support the predictive value of β-synuclein as suggested in previous cross-sectional studies [1–4]. It could therefore be used as a marker for patient stratification in clinical trials or decision-making in disease monitoring but needs further confirmation. During the preclinical and early symptomatic phase of sporadic AD, our data indicate slowly increasing β-synuclein levels which is in contrast to our observation in ADAD, where β-synuclein levels were already increased in asymptomatic AD mutation carriers [6]. Similar findings were reported for Down syndrome [5]. This could point to different trajectories of β-synuclein in sporadic and genetic forms of AD. However, a limitation of our study is that it is based on clinical diagnosis of participants, including also some Aβ-negative cases and a substantial number of individuals with unknown Aβ status (discussed in more detail below). This could especially affect our observations in this early phase where first symptoms might not be specific enough for AD and needs further verification.

In AD dementia patients, our findings in follow-up samples and temporal staging of patients in the AD continuum support slow longitudinal changes of serum β-synuclein in this phase. On the other hand, we observed higher β-synuclein levels in moderate vs mild dementia indicating the existence of a stage-dependent increase. However, this is not necessarily a conflicting observation and can be explained by the observed RoC in AD dementia and the time difference between mild and moderate AD that might occur, especially since we don´t know how long those patients already belong to these stages. We here also compared serum β-synuclein with CSF NRGN and observed a significant correlation of both markers. Overall, β-synuclein outperformed NRGN in the discrimination of AD dementia and MCI patients, Aβ+ and Aβ- subjects and in the association with cognitive scores. This needs further confirmation but it further supports β-synuclein as a promising read-out for clinical trials.

A limitation of our study is the selection and group allocation of participants mainly by clinical diagnosis. The participants investigated here were recruited in the first study phase of ADNI providing us clinical diagnostic follow-up data for up to 19 years enabling exact staging of participants within the AD continuum. However, at this phase of ADNI, no amyloid PET scans were available to support amyloid pathology. CSF Aβ42 levels were available for a subset of participants only but these data uncoverred several Aβ- subjects in the MCI group. Thus, especially early MCI and CU individuals might not be correctly assigned to the AD continuum. Our observations should therefore be verified in a cohort with biologically and clinically defined AD stages. In addition, data on other blood and CSF biomarkers such as pTau217, pTau181 or GFAP were not available for our samples which is why we could not compare and evaluate the observations for β-synuclein in relation to these biomarkers.

## Conclusions

In conclusion, our longitudinal data support the use of serum β-synuclein levels for prediction of future cognitive decline and MCI-to-dementia conversion which should be confirmed in future studies. Further studies with biologically and clinically defined participants and larger sample size must verify the trajectories of β-synuclein in the preclinical and early MCI phase. Follow-up samples over a longer timeframe should resolve the exact time point of the β-synuclein increase. In addition, more studies on potential confounders for blood β-synuclein are required to reduce inter-individual variation of values.

## Supplementary Information

Supplementary Material 1: Supplement Fig. S1. Overview on participants included in the study and availability of serum samples and clinical follow-up data.

## Data Availability

The datasets generated during and/or analysed during the current study are available from the ADNI database (adni.loni.usc.edu).

## Abbreviations

Aβ+: Aβ-positivity
AD: Alzheimer´s disease
ADAD: Asymptomatic autosomal dominant AD
ADNI: Alzheimer’s Disease Neuroimaging Initiative
AUC: Area under the curve
CDR-SB: Clinical dementia rating sum-of-boxes
CU: Cognitively unimpaired
DIAN: Dominantly Inherited Alzheimer Network
IQR: Interquartile range
MCI: Mild cognitive impairment
MMSE: Mini-Mental State Examination
MRI: Magnetic resonance imaging
MRM: Mutilpe reaction monitoring
NfL: Neurofilament light chain
NRGN: Neurogranin
PET: Positron emission tomography
pTau181: Tau protein phosphorylated at S181
QC: Quality control
RoC: Rate-of-change
ROC: Receiver operating characteristic
tTau: Total Tau
