# Supplementary material for: Longitudinal changes of blood β-synuclein in cognitively unimpaired, mild cognitive impairment and sporadic Alzheimer´s disease
Source: Alzheimers Res Ther. 2026 Feb 11;18:45. doi: 10.1186/s13195-026-01973-1 (PMC12930813; doi:10.1186/s13195-026-01973-1)
Supplement: Supplementary file 1 — Supplementary Material 1: Supplement Fig. S1. Overview on participants included in the study and availability of serum samples and clinical follow-up data. [file 13195_2026_1973_MOESM1_ESM.pdf]

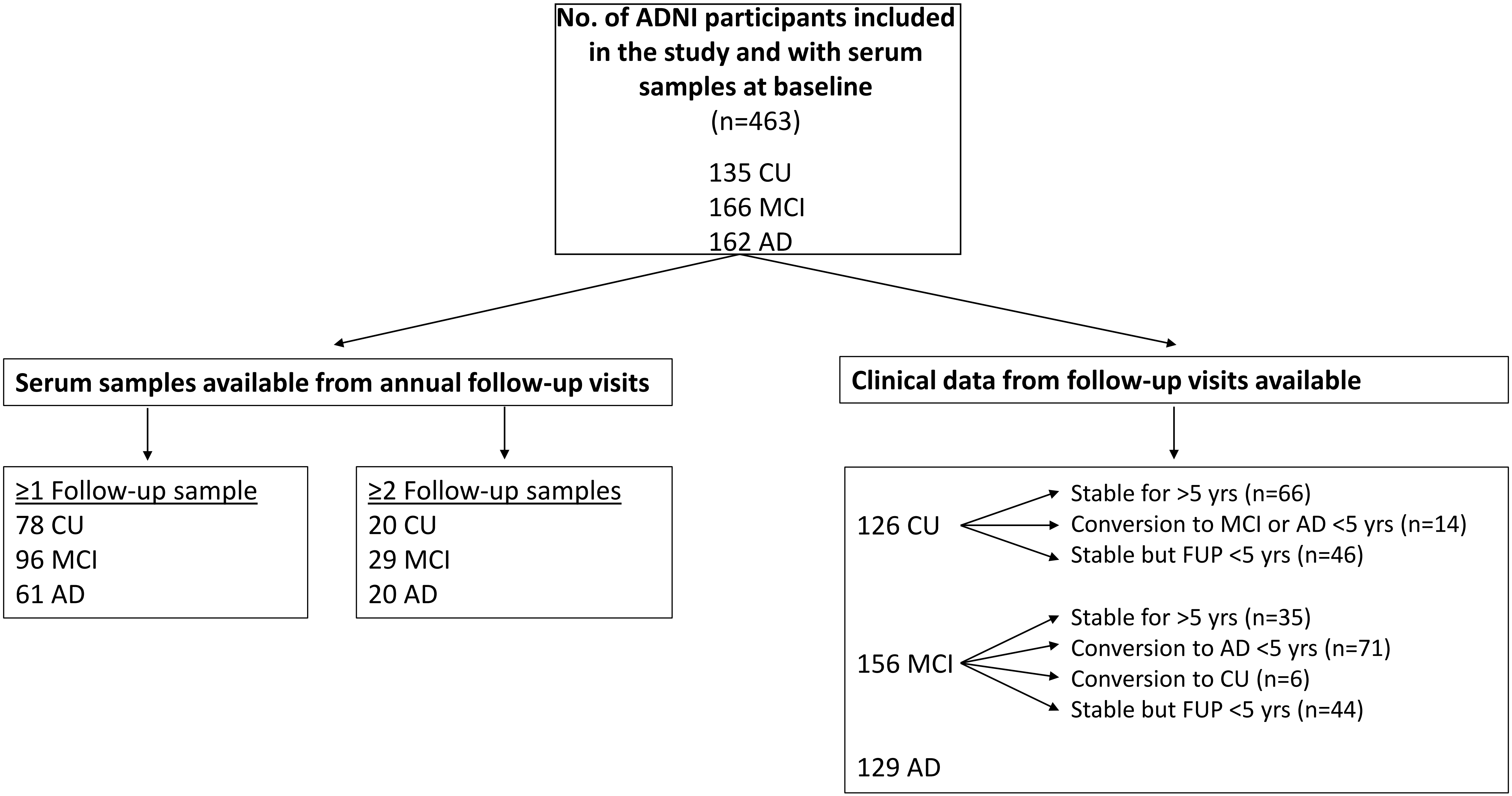
No. of ADNI participants included in the study and with serum samples at baseline
(n=463)
135 CU
166 MCI
162 AD
Serum samples available from annual follow-up visits
≥1 Follow-up sample
78 CU
96 MCI
61 AD
≥2 Follow-up samples
20 CU
29 MCI
20 AD
Clinical data from follow-up visits available
126 CU
Stable for >5 yrs (n=66)
Conversion to MCI or AD <5 yrs (n=14)
Stable but FUP <5 yrs (n=46)
156 MCI
Stable for >5 yrs (n=35)
Conversion to AD <5 yrs (n=71)
Conversion to CU (n=6)
Stable but FUP <5 yrs (n=44)
129 AD
